# Integrated ATAC-Seq and RNA-Seq Identify ATF3 as a Tumor Suppressor in UVB-Induced Keratinocyte Carcinogenesis

**DOI:** 10.3390/biology15141101

**Published:** 2026-07-08

**Authors:** Zhi Guo, Xuyi Deng, Sheng Lu, Fenghao Liu, Meijuan Zhou, Yinghui Wang

**Affiliations:** Department of Radiation Medicine, Guangdong Provincial Key Laboratory of Tropical Disease Research, School of Public Health, Southern Medical University, Guangzhou 510515, China; guoz0405@gmail.com (Z.G.); dengxuyi@smu.edu.cn (X.D.); ls804469506@163.com (S.L.); puddingxlarry@163.com (F.L.)

**Keywords:** ATAC-seq, RNA-seq, chronic UVB radiation, keratinocyte carcinogenesis, ATF3

## Abstract

Current approaches for preventing and suppressing the initiation and development of keratinocyte carcinoma remain limited, highlighting the urgent need to identify novel targets for tumor intervention. As major environmental pathogenic factor, chronic ultraviolet B (UVB) exposure promotes keratinocyte malignant transformation through epigenetic reprogramming, reshaping chromatin accessibility and gene expression program, which has not been fully elucidated. Here, we established a chronic UVB irradiation model to induce malignant transformation of keratinocytes and performed integrative analyses using ATAC-seq and RNA-seq. We identified 4903 differentially accessible regions (DARs), with increased accessibility at loci of tumor-promoting genes such as *TP63*, *KRT13*, *KLF8*, and *EGFR*, and enrichment of AP-1 family motifs. RNA-seq revealed 1136 differentially expressed genes (DEGs), predominantly enriched in inflammatory pathways. Integrative analysis highlighted concordant enrichment of inflammation-related genes within open chromatin regions. ATF3 was selected and its downregulated identified after UVB exposure. Functional assays confirmed that ATF3 represses chronic UVB-induced malignant transformation of keratinocytes. Notably, ChIP-qPCR validation verified *GDA* (Guanine deaminase) as the direct downstream target gene of ATF3. Reduced ATF3 binding elevates *GDA* promoter chromatin accessibility and upregulates GDA expression to drive malignant progression. In conclusion, our findings demonstrate that chronic UVB exposure remodels chromatin and transcriptomic landscapes and identify ATF3 and its direct target *GDA* as a critical tumor-suppressive regulatory module for UVB-induced keratinocyte carcinoma.

## Simple Summary

Chronic ultraviolet radiation B (UVB) exposure is a major environmental driver of keratinocyte carcinoma, which promotes skin tumorigenesis through epigenetic and transcriptional dysregulation. The molecular mechanism connecting chromatin remodeling, inflammatory activation and keratinocyte malignant transformation remains poorly clarified. Here, integrated ATAC-seq and RNA-seq were combined to systematically profile genome-wide chromatin accessibility and transcriptomic alterations under continuous UVB stimulation. Chromatin regions with enhanced accessibility were enriched in oncogenic genes, while differentially expressed genes primarily clustered in inflammation-related pathways. Motif analysis revealed prominent enrichment of the AP-1 transcription factor family, and ATF3 was further identified as a core regulatory molecule during malignant progression. Functional experiments verified that ATF3 suppresses chronic UVB-induced malignant behaviors. This study delineates a critical mechanism underlying chronic UVB-driven skin carcinogenesis, and provides a novel tumor suppressor and promising therapeutic target for the prevention and intervention of keratinocyte carcinoma.

## 1. Introduction

Keratinocyte carcinoma represents one of the most prevalent non-melanoma skin cancers (NMSCs), accounting for approximately 20–30% of all skin cancer cases globally, with a steadily rising annual incidence [1]. Epidemiological and molecular evidence has firmly established that chronic ultraviolet B (UVB, 280–315 nm) irradiation serves as the primary environmental risk factor for the development of keratinocyte carcinoma [2]. As the outermost protective barrier of the human body, the skin is directly exposed to UVB radiation, which penetrates the epidermis and causes cumulative damage to keratinocytes, the predominant cellular component of the epidermis [3]. Despite widespread implementation of sun protection strategies, their efficacy in reducing the incidence of keratinocyte carcinoma remains limited. This underscores the urgent need to elucidate the molecular mechanisms underlying UVB-induced keratinocyte carcinoma and identify novel preventive and therapeutic targets.

The malignant transformation of normal keratinocytes driven by chronic UVB irradiation is a multistep progressive process encompassing inflammation, precancerous lesions, and malignant progression to invasive keratinocyte carcinoma [4,5]. Dynamic changes in chromatin accessibility and transcription factor binding occur throughout the entire oncogenic process. UVB continuous exposure elicits reactive oxygen species (ROS) production, oxidative stress, and inflammation, which contributes to the epigenetic aberrations [6]. The dysregulation of transcription factors coupled with altered chromatin dynamics are crucial for cutaneous squamous cell carcinoma pathogenesis. Among these regulators, ΔNp63α, the dominant TP63 isoform, promotes SCC progression by bypassing oncogene-induced senescence and synergizing with oncogenic Ras [7]. Chronic UVB irradiation drives malignant transformation through aberrant EGFR activation, enhancing keratinocyte proliferation and conferring chemo- and radio-resistance [8]. Additionally, KLF8 facilitates invasion and metastasis via epithelial–mesenchymal transition (EMT) [9], while KRT13 downregulation disrupts epithelial integrity [10], collectively driving cutaneous malignancy. Therefore, understanding the epigenetic and transcriptional mechanisms underlying this process is essential.

ATAC-seq efficiently identifies genome-wide open chromatin regions, which are critical for transcription factor binding and transcriptional regulation, providing insights into epigenetic reprogramming [11,12]. Combined with RNA-seq, this approach will profile global gene expression patterns and elucidate UVB-driven oncogenic reprogramming, clarify the crosstalk between chromatin dynamics and transcriptional regulation, and identify key molecular targets that mediate keratinocyte malignant transformation. Activating transcription factor 3 (ATF3), a member of the ATF/CREB family of basic-region leucine zipper (bZIP) transcription factors, is a stress-responsive gene that plays a pivotal role in tumorigenesis [13]. Cumulative evidence has firmly established that ATF3 exerts dual context-dependent functions in tumorigenesis. It can act as a tumor suppressor by transcriptionally repressing diverse oncogenic programs, such as MYCN-driven proliferation, BCL2L1-mediated survival, SLC7A11-dependent ferroptosis resistance, MGMT-mediated DNA repair, and metastasis-related genes including IFI6, IFI27, and MMP2 [14,15]. Conversely, ATF3 may also facilitate keratinocyte tumorigenesis under specific conditions, for example via repressing p53 expression to activate Stat3 phosphorylation and blocking calcineurin/NFAT-induced p53-dependent cellular senescence [16,17]. Given such divergent roles, the exact transcriptional function of ATF3 in chronic UVB-induced malignant transformation of keratinocytes remains to be elucidated.

Here, we constructed the in vitro model of chronic UVB-induced keratinocyte malignant transformation and employed integrated ATAC-seq and mRNA-seq analyses to comprehensively profile chromatin accessibility and transcriptome dynamics. Screening results pinpointed ATF3 as the critical transcription factor regulating UVB-triggered keratinocyte carcinogenesis. Chronic UVB irradiation suppresses ATF3 expression, which augments chromatin accessibility of its target gene *GDA* and alleviates of transcriptional repression on GDA, ultimately driving UVB-induced malignant transformation of keratinocytes. This study clarifies the epigenetic and transcriptional alterations involved in the keratinocyte tumorigenesis triggered by UVB irradiation as well as the definite tumor suppressive role of ATF3, providing potential targets for the prevention and early intervention of keratinocyte carcinoma.

## 2. Materials and Methods

### 2.1. Cell Culture and UVB Irradiation

Immortalized human keratinocyte cell line HaCaT (RRID:CVCL_0038, HonSun Biological Technology Co., Ltd., Shanghai, China), cutaneous squamous cell carcinoma cell line HSC-1 (RRID:CVCL_2807, HonSun Biological Technology Co., Ltd., Shanghai, China) and HSC-5 (RRID:CVCL_0314, HonSun Biological Technology Co., Ltd., Shanghai, China) were cultured in DMEM (Gibco, Grand Island, NY, USA) supplemented with 10% fetal bovine serum (FBS, Gibco, Grand Island, NY, USA) and 1% penicillin-streptomycin (Gibco, Grand Island, NY, USA) at 37 °C in a humidified atmosphere containing 5% CO_2_. All cell lines were authenticated using short tandem repeat (STR) profiling within the last 3 years. All experiments were performed with mycoplasma-free cells. For chronic UVB-induced malignant transformation, HaCaT cells were subjected to repeated UVB irradiation (312 nm, Sankyo Co., Ltd., Tokyo, Japan) at a dosage of 30 J/m^2^ once weekly for a total duration of 16 consecutive weeks. During the entire irradiation cycle, cells were routinely passaged under standard culture conditions to maintain the logarithmic growth phase, and culture medium was refreshed regularly to ensure cell viability. Cells in the control group were cultured in parallel without UVB exposure, with identical passaging and culture protocols applied to maintain consistency.

### 2.2. ATAC-Seq Library Construction and Sequencing

ATAC-seq was performed following published procedures with minor modifications [18]. Briefly, 5 × 10^5^ control and UVB-irradiated HaCaT cells were collected and washed twice with cold phosphate-buffered saline (PBS). Cells were lysed with lysis buffer (10 mM Tris-HCl pH 7.4, 10 mM NaCl, 3 mM MgCl_2_, 0.1% IGEPAL CA-630) on ice for 10 min. Nuclei were collected by centrifugation at 500× *g* for 10 min at 4 °C and resuspended in transposition reaction mix containing Tn5 transposase (Illumina, San Diego, CA, USA). The transposition reaction was carried out at 37 °C for 30 min. Transposed DNA was purified using a MinElute PCR Purification Kit (Qiagen, Hilden, Germany). Library construction was performed by PCR amplification with index primers, and the library quality was assessed using an Agilent 2100 Bioanalyzer (Agilent Technologies, Santa Clara, CA, USA). Sequencing was performed on an Illumina NovaSeq 6000 platform (Illumina, Inc., San Diego, CA, USA) with 150 bp paired-end reads.

### 2.3. RNA-Seq Library Construction and Sequencing

Total RNA was extracted from control and UVB-irradiated HaCaT cells using TRIzol reagent (Invitrogen, Carlsbad, CA, USA) according to the manufacturer’s instructions. RNA quality was evaluated using a NanoDrop 2000 spectrophotometer (Thermo Fisher Scientific, Waltham, MA, USA) and Agilent 2100 Bioanalyzer (Agilent Technologies, Santa Clara, CA, USA). mRNA was enriched using oligo(dT) magnetic beads, and fragmented into small pieces. First-strand cDNA was synthesized using random hexamer primers, followed by second-strand cDNA synthesis. The cDNA library was constructed using the VAHTS Universal V6 RNA-seq Library Prep Kit for Illumina (Vazyme Biotech Co., Ltd., Nanjing, China) through a series of procedures including end repair, A-tailing, adapter ligation, and PCR amplification. After strict quality verification of the constructed libraries, high-throughput transcriptome sequencing was performed on the Illumina NovaSeq 6000 platform (Illumina, Inc., San Diego, CA, USA) with 150 bp paired-end reads. All RNA-seq library construction and sequencing services were commercially outsourced to Wuhan Kang ce Technology Co., Ltd. (Wuhan, China).

### 2.4. Bioinformatics Analysis

For ATAC-seq data analysis: Raw reads were filtered to remove low-quality reads and adapters using Trimmomatic (v0.39). Clean reads were aligned to the human genome (hg38) using Bowtie2 (v2.5.4). Duplicate reads were removed using Picard Tools. Peak calling was performed using MACS2 (v2.2.9.1) with a q-value cutoff of 0.05. Herein, q-value represents the false discovery rate (FDR) after multiple testing correction, which serves as the core screening threshold for identifying statistically significant accessible peaks, differential chromatin regions and functional enrichment pathways. Differential accessibility peaks between control and UVB-irradiated groups were identified using DESeq2 (v1.38.3) with |log2(fold change)| > 1 and adjusted *p*-value < 0.05. Motif analysis of differential peaks was performed using HOMER (v24.05.1) to predict potential transcription factors.

For RNA-seq data analysis: Raw reads were filtered using Trimmomatic. Clean reads were aligned to the human genome (hg38) using Hisat2 (v2.1.0). Gene expression levels were calculated using StringTie (v2.2.1) as fragments per kilobase of transcript per million mapped reads (FPKM). Differential expressed genes (DEGs) were identified using DESeq2 with |log2(fold change)| > 1 and adjusted *p*-value < 0.05. Functional enrichment analysis of DEGs, including Gene Ontology (GO) and Kyoto Encyclopedia of Genes and Genomes (KEGG) pathway analysis, was performed using clusterProfiler (v4.12.0).

Putative downstream target genes of transcription factors were predicted using the ChEA Transcription Factor Targets Dataset (https://maayanlab.cloud/Harmonizome/dataset/ChEA+Transcription+Factor+Targets, accessed on 11 September 2025). This database compiles high-confidence TF-target gene pairs primarily curated from published ChIP-seq chromatin binding experiments. Briefly, we retrieved all annotated target genes assigned to ATF3 within experimentally validated ChIP-seq libraries including ENCODE ChIP-seq (ENCODE Data Coordinating Center, Stanford, CA, USA, https://www.encodeproject.org/, accessed on 11 September 2025).and Literature ChIP-seq (Ma’ayan Laboratory, Icahn School of Medicine at Mount Sinai, NY, USA, https://maayanlab.cloud/Harmonizome/dataset/ChEA+Transcription+Factor+Targets, accessed on 11 September 2025). All ATF3-associated target entries from chromatin-binding experimental libraries were downloaded as TSV files, followed by merging and deduplication to generate a high-confidence list of ATF3 downstream candidate targets.

### 2.5. Plasmid Construction and Cell Transfection

The full-length coding sequence of ATF3 was cloned into the pcDNA3.1 (+) vector (Invitrogen, Carlsbad, CA, USA) to construct the ATF3 overexpression plasmid (pcDNA3.1-ATF3). Small interfering RNAs (siRNAs) targeting ATF3 (si-ATF3-1: 5′-GGAUUAUCCUGAAGAAGAATT-3′; si-ATF3-2: 5′-CCAUUUGCUGUGAUGAAGATT-3′; si-ATF3-3: 5′-CGAGAAGCAGCAUUUGAUA-3′) and negative control siRNA (si-NC: 5′-UUCUCCGAACGUGUCACGUTT-3′) were purchased from GenePharma (Shanghai, China).

UVB-irradiated HaCaT cells were seeded in 6-well plates at a density of 2 × 10^5^ cells per well. When cells reached 70–80% confluence, transfection was performed using Lipofectamine 3000 reagent (Invitrogen, Carlsbad, CA, USA) according to the manufacturer’s instructions. At 48 h post-transfection, cells were collected for subsequent experiments.

### 2.6. CCK-8 Assay

Cell proliferation ability was detected using the Cell Counting Kit-8 (CCK-8, Dojindo, Kumamoto, Japan). Transfected cells were seeded in 96-well plates at a density of 2 × 10 cells per well. At 0, 24, 48, and 72 h post-seeding, 10 μL of CCK-8 solution was added to each well and incubated at 37 °C for 2 h. The absorbance at 450 nm was measured using a microplate reader. Each experiment was performed in triplicate.

### 2.7. Colony Formation Assay

Transfected cells were seeded in 6-well plates at a density of 500 cells per well and cultured at 37 °C for 14 days. The medium was changed every 3 days. After 14 days, colonies were fixed with 4% paraformaldehyde for 30 min and stained with 0.1% crystal violet for 20 min. Colonies with more than 50 cells were counted under a microscope. The colony formation rate was calculated as (number of colonies/number of seeded cells) × 100%. Each experiment was performed in triplicate.

### 2.8. Soft Agar Colony Formation Assay

Anchorage-independent growth was assessed via soft agar colony formation assay. A base layer containing 0.6% low-melting agarose in complete DMEM was solidified in 6-well plates, followed by a top layer mixed with 0.3% low-melting agarose and 5 × 10^3^ control or chronic UVB-irradiated HaCaT cells. After 21 days of incubation at 37 °C, colonies were fixed and imaged. Colonies over 50 μm in diameter were quantified, with three independent biological replicates conducted for statistical validation.

### 2.9. Transwell Migration Assays

Transfected cells were resuspended in serum-free DMEM. A total of 2 × 10^5^ cells were added to the upper chamber of a Transwell insert (8 μm pore size, Corning, NY, USA). DMEM containing 10% FBS was added to the lower chamber. After incubation at 37 °C for 24 h, cells on the upper surface of the insert were removed with a cotton swab. Cells that migrated to the lower surface were fixed with 4% paraformaldehyde and stained with 0.1% crystal violet. The number of migrated cells was counted in five random fields under a microscope.

### 2.10. Quantitative Real-Time PCR

Total RNA was extracted from HaCaT and cSCC cells using Trizol reagent (Invitrogen, Carlsbad, CA, USA). The concentration and purity of extracted RNA were (260/280 ratio) determined using NanoDrop (Thermo Fisher Scientific Inc., Waltham, MA, USA), and cDNA was reverse-transcribed using the Evo M-MLV RT Kit for qPCR (Accurate biology, Guangzhou, China). qPCR was performed with 2 × Taq SYBR Green qPCR Mix (Vazyme, Nanjing, China) on QuantStudio 6 (Thermo Fisher Scientific Inc., Waltham, MA, USA), using GAPDH as internal reference. Primer sequences used are listed in Table 1, and relative expression was calculated via the 2^−ΔΔCt^ method in triplicate.

### 2.11. Western Blot Assay and Immunofluorescence Staining

For Western blotting, total proteins were extracted from the cells by homogenizing them in RIPA buffer with the addition of protease and phosphatase inhibitor. The concentration of protein in the cell lysate was determined using the Bradford Protein Assay (Bio-Rad Laboratories, Inc., Hercules, CA, USA). Equal amounts of protein were separated by 10% SDS-polyacrylamide gel electrophoresis, then blotted onto polyvinylidene fluoride (PVDF) membranes (Millipore, Burlington, MA, USA). The membranes were blocked using 5% non-fat milk to prevent non-specific binding, incubated overnight at 4 °C with primary antibodies against ATF3 (1:1000, 18665, Cell Signaling Technology, Danvers, MA, USA), E-cadherin (1:1000, 20874-1-AP, Proteintech Group, Inc., Rosemont, IL, USA), N-cadherin (1:1000, 22018-1-AP, Proteintech Group, Inc., Rosemont, IL, USA), Vimentin (1:20,000, 10366-1-AP, Proteintech Group, Inc., Rosemont, IL, USA), Snail (1:1000, 3879, Cell Signaling Technology, Danvers, MA, USA), Slug (1:1000, 9585, Cell Signaling Technology, Danvers, MA, USA), p65 (1:5000, R380172, ZenBio, Inc., Durham, NC, USA), p-p65(Ser536) (1:1000, 310013, ZenBio, Inc., Durham, NC, USA), IKBα(R380682, ZenBio, Inc., Durham, NC, USA), p-IKB α(Ser32/Ser36) (1:1000, 340776, ZenBio, Inc., Durham, NC, USA) and mouse anti-rabbit β-actin (1:2000, Santa Cruz Biotechnology, Inc., Dallas, TX, USA). Subsequently, the membranes were washed three times with Tris-buffered saline supplemented with Tween20 (TBST), incubated with HRP-conjugated goat anti-rabbit IgG (1:4000, 7074, Cell Signaling Technology, Danvers, MA, USA) and anti-rabbit IgG (1:4000, 7076, Cell Signaling Technology, Danvers, MA, USA) for 1 h at room temperature, followed visualizing by an enhanced chemiluminescence system. All uncropped original Western blot images are provided in Supplementary File S1.

For immunofluorescence staining, the transfected cells were seeded on sterilized glass coverslips in 24-well plate. After irradiation, the cells were fixed with 4% paraformaldehyde in PBS for 10 min. Next, the cells were blocked in the block buffer composed of Triton X-100, PBS and goat serum for 1 h. Then incubated overnight at 4 °C with primary antibodies against γH2A.X (1:500, 9718S, Cell Signaling Technology, Danvers, MA, USA) diluted in antibody buffer contained PBS, Triton X-100 and BSA, followed by incubation with the appropriate fluorescently labeled anti-goat secondary antibody (1:250, SA00013-4, Proteintech Group, Inc., Rosemont, IL, USA). The cell nuclei were stained with DAPI (Beyotime, Shanghai, China). Images were acquired under an inverted fluorescence microscope (Carl Zeiss AG, Oberkochen, Germany). The number of γH2A.X foci per cell were identified and counted via the threshold adjustment and particle analysis function of Image J (v1.54t), with unified particle size and circularity thresholds set for all samples. At least 100 cells were randomly quantified for each group from three independent biological replicates.

### 2.12. Chromatin Immunoprecipitation Coupled with Quantitative Real-Time PCR (ChIP-qPCR) Assay

ChIP-qPCR was implemented to detect the binding of ATF3 to the promoter sequences of *DKK1*, *GDA*, *CARD16* and *TFPI2*. Briefly, cells were cross-linked with 1% formaldehyde at 37 °C for 10 min, followed by quenching with 125 mM glycine at room temperature for 5 min. Cells were washed with cold PBS and lysed in buffer containing 1% SDS, 1 mM EDTA, and 25 mM Tris-HCl (pH 8.0) for 30 min at 4 °C. Supernatant was collected after centrifugation and sonicated to shear chromatin into 100–500 bp fragments. Chromatin samples were immunoprecipitated using ChIP-validated anti-ATF3 antibody, with normal rabbit IgG set as negative control and unprocessed chromatin as input reference. Protein–DNA complexes were washed sequentially with low-salt, high-salt, LiCl, and TE buffers at 4 °C. Elution was performed with 1% SDS and 0.1 M NaHCO_3_ at 37 °C for 30 min. Reverse crosslinking was achieved by adding NaCl (0.2 M) and proteinase K (0.5 mg/mL) and incubating at 65 °C overnight. DNA was purified using a DNA purification kit, dissolved in ddH_2_O, qPCR was conducted with primers specific for ATF3-binding promoter regions. All ChIP-qPCR primers were designed with Primer Premier 6.0 software, and their specificity was verified via NCBI BLAST analysis (https://blast.ncbi.nlm.nih.gov/Blast.cgi, accessed on 28 May 2026). Sequences of all utilized primers are summarized in Table 2. Relative enrichment fold changes were quantified to evaluate ATF3 occupancy on target gene promoters, with three independent biological replicates included for statistical analysis.

### 2.13. Statistical Analysis

Data are presented as mean ± standard deviation (SD) from at least three independent experiments. Statistical comparisons between two groups were performed using two-tailed Student’ s *t*-test. One-way analysis of variance (ANOVA) followed by Tukey’ s post hoc test was used for multiple group comparisons. A *p*-value < 0.05 was considered statistically significant. For ATAC-seq and RNA-seq, differentially expressed genes and differentially accessible regions were identified with adjusted *p*-value < 0.05 and fold change > 1.5 as cutoff thresholds. Motif and transcription factor enrichment analyses were evaluated with statistically significant enrichment scores and *p*-values.

## 3. Results

### 3.1. Chronic UVB-Induced Keratinocyte Malignant Transformation

**Figure 1:**
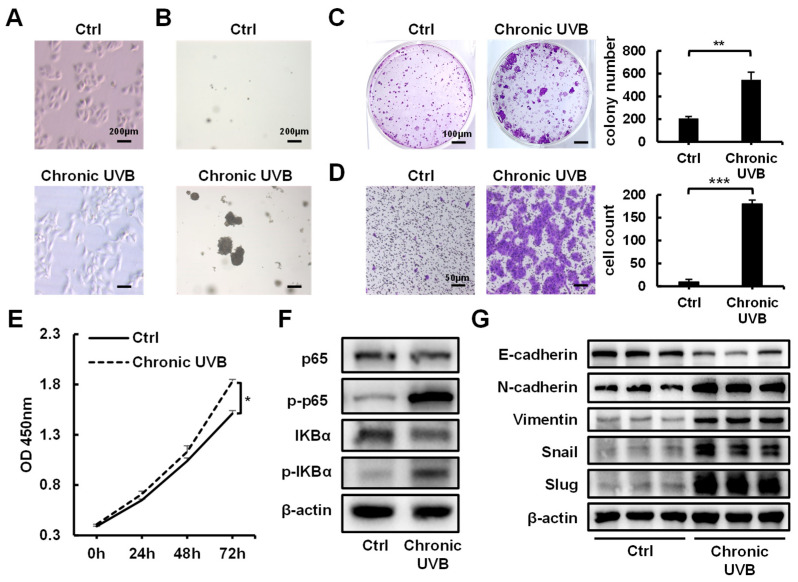
Chronic UVB irradiation drives malignant transformation of HaCaT keratinocytes. (**A**) Morphological changes in control (Ctrl) and chronic UVB-irradiated HaCaT (Chronic UVB) cells. (**B**) Soft agar assay for anchorage-independent growth of normal and UVB-transformed keratinocytes. (**C**) Colony formation assay demonstrates enhanced clonogenicity in UVB-irradiated HaCaT cells. (**D**) Transwell migration assay reveals increased migratory potential of UVB-irradiated HaCaT cells. (**E**) CCK-8 assay shows increased proliferation of UVB-irradiated cells compared with control (*n* = 3, * *p* < 0.05). (**F**) Western blot analysis of NF-κB pathway components p65, p-p65, IκBα and p-IκBα; β-actin serves as loading control. (**G**) Immunoblotting detection of EMT signature proteins E-cadherin, N-cadherin, Vimentin, Snail and Slug; β-actin served as internal reference. * *p* < 0.05, ** *p* < 0.01, *** *p* < 0.001.

To simulate chronic, cumulative, and non-acute lethal photodamage in vitro and effectively induce the malignant transformation of human keratinocytes, HaCaT cells were subjected to UVB irradiation at a dose of 30 J/m^2^ once weekly for 16 consecutive weeks. This protocol closely recapitulates the pathophysiological process of long-term ultraviolet light exposure in human skin in vivo. Following completion of the chronic irradiation procedure, chronic UVB-irradiated HaCaT cells displayed markedly distinct cellular morphology relative to unirradited HaCaT cells, with cells undergoing malignant morphological transformation characterized by a spindle-shaped and irregular appearance (Figure 1A). Multiple functional assays were conducted to characterize the oncogenic phenotypes of UVB-irradiated HaCaT cells. Soft agar assays, a gold standard for anchorage-independent tumorigenic growth, showed rare tiny aggregates in controls but abundant large dense colonies after chronic UVB irradiation (Figure 1B). Meanwhile, plate colony formation assay further verified that long-term UVB irradiation drastically boosted the clonogenic survival capacity of HaCaT cells (Figure 1C). Transwell migration assay demonstrated that sustained UVB stimulation conferred migratory potential to HaCaT cells, a phenotype barely observed in unirradited HaCaT cells (Figure 1D). Consistently, CCK-8 proliferation assay revealed significantly elevated proliferative activity in chronic UVB groups, with prominent differences observed at 72 h post cell seeding (Figure 1E). Mechanistically, immunoblotting of NF-κB signaling components uncovered unchanged total p65, yet prominent upregulation of p-p65 and p-IκBα, alongside reduced total IκBα in chronic UVB cells, demonstrating robust NF-κB signaling activation upon chronic UVB irradiation (Figure 1F). Epithelial–mesenchymal transition (EMT), a core program driving tumor migration and invasion, was additionally validated at the protein level via Western blot (Figure 1G). Compared with control cells, chronic UVB treatment markedly downregulated the epithelial marker E-cadherin. By contrast, the mesenchymal markers N-cadherin and Vimentin, as well as EMT transcription factors Snail and Slug, were substantially upregulated after long-term UVB irradiation. Overall, these phenotypic, functional and molecular results demonstrate that chronic UVB irradiation successfully induces malignant transformation in HaCaT keratinocytes.

### 3.2. Continuous UVB Irradiation-Altered Chromatin Accessibility

**Figure 2:**
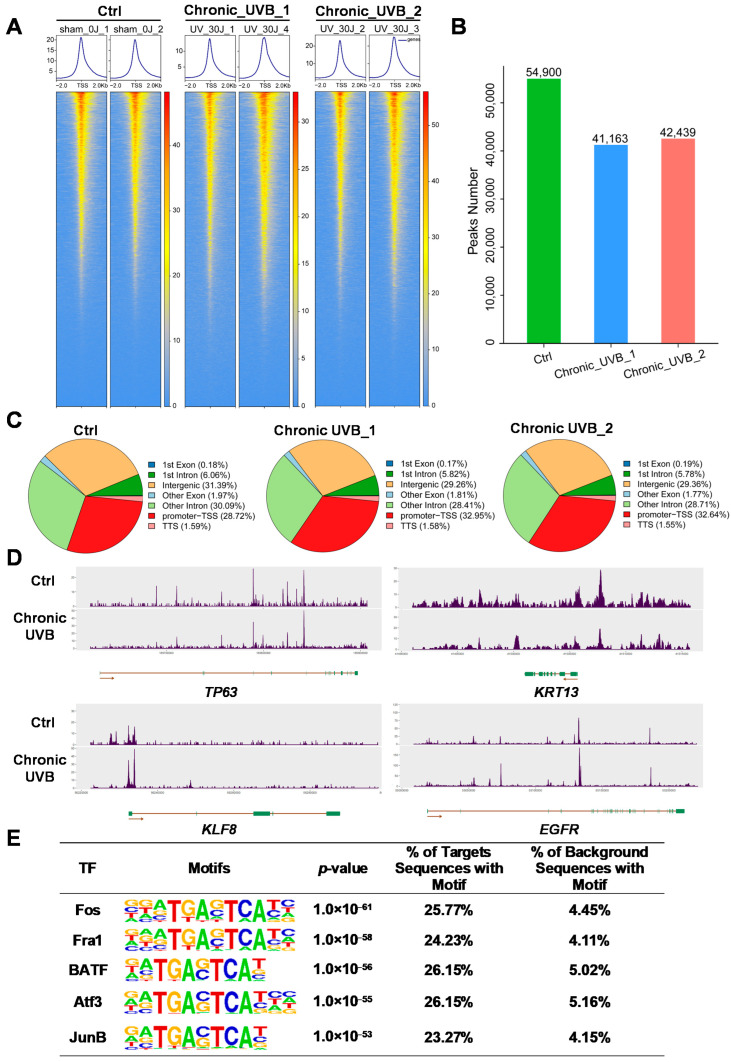
Chromatin accessibility changes and motif enrichment in chronic UVB-irradiated HaCaT cells. (**A**) Metagene plots of ATAC-seq reads around TSS in control and chronic UVB groups. (**B**) Peak count comparison between control and chronic UVB groups. (**C**) Genomic distribution of ATAC-seq peaks, showing predominant enrichment in promoter–TSS regions. (**D**) Genome tracks showing accessibility changes at *TP63*, *KRT13*, *KLF8*, *EGFR* loci. Purple peaks: ATF3 ChIP-seq read coverage signals of two groups; Green blocks: Exon regions of genes; Brown arrow: Transcriptional direction of genes. (**E**) Top enriched transcription factor motifs from DARs, highlighting significant enrichment of AP-1 family members including Atf3.

In order to investigate genome-wide chromatin accessibility changes in normal keratinocytes after chronic UVB irradiation, ATAC-seq was performed on unirradiated or chronic UVB-irradiated HaCaT cells. The sequencing coverage and quality statistics of ATAC-seq data are summarized in Supplementary File S2. A total of 439,573,882 raw reads were generated, yielding 429,376,812 clean reads with a Q30 value > 93.9% (Table S1), indicating high sequencing quality and suitability for subsequent analyses. Peak analysis revealed that the identified peaks were predominantly concentrated within 2 kb upstream and downstream of genes, suggesting that these chromatin accessible regions are involved in transcriptional regulation (Figure 2A). The hierarchical clustering heat map of DARs showed consistent expression of peaks within the groups (Figure S1A), verifying the reproducibility and usability of the sequencing data.

A total of 138,502 ATAC-seq peaks were identified in the samples from unirradiated and chronic UVB-irradiated HaCaT cells, which were distributed on each chromosome (Figure S1B). Genomic distribution and peak number analysis revealed that unirradiated HaCaT cells harbored more peaks enriched in intergenic and intronic regions, whereas chronic UVB irradiation increased the promoter-associated peaks although it reduced the peak number (Figure 2B,C). It demonstrates that chronic UVB exposure induces a global shift from widespread, distal element-driven open chromatin to a focused, promoter-centered accessibility pattern. This alteration would facilitate the robust transcriptional activation of oncogenic and stress-response genes during UVB-induced malignant transformation.

To identify genes exhibiting chromatin opening under chronic UVB irradiation, we examined accessibility changes at functionally relevant loci. Compared with the non-irradiated cells, the chronic UVB-irradiated cells showed significantly increased chromatin accessibility peak intensities for *TP63*, *EGFR* and *KLF8*, while decreased for *KRT13* (Figure 2D). These findings suggest that chronic UVB irradiation remodels the chromatin landscape to activate pro-tumorigenic transcription factors and oncogenes while suppressing epithelial differentiation markers, thereby priming keratinocytes for malignant transformation.

In addition, motif prediction and enrichment analysis for DARs showed 91 transcription factors were significantly enriched. Among those, the top five transcription factors annotated by DARs were c-Fos, Fra1, BATF, ATF3 and JUNB (Figure 2E). These transcription factors are all core members of the AP-1 protein complex or its interacting partners. Notably, whereas most identified AP-1 family members act as oncogenic drivers in keratinocyte carcinoma, ATF3 stands out as a unique transcription factor with reported tumor-suppressive functions, making it a high-priority candidate for mechanistic investigation. c-Fos heterodimerizes with JunB to regulate cell proliferation, EMT and matrix degradation, which exerts pro-tumor effects and promotes cSCC progression [19]. Fra1, frequently overexpressed in cSCC tissues, facilitates invasion and metastasis through EMT and Matrix metalloproteinases (MMPs) [20]. BATF forms heterodimers with JunB to remodel tumor microenvironment and promote cSCC by regulating the expression of IL-6 and IL-8 [21]. ATF3 suppresses cSCC by dysregulating p53-dependent senescence pathways, and its downregulation activates cancer-associated fibroblasts (CAFs) thereby promotes cSCC progression [22]. JunB sustains epidermal homeostasis normally, while its dysregulation in cSCC promotes proliferation, and modulates the tumor immune microenvironment [23]. Specifically, 26.15% of target DAR sequences contained the ATF3 motif, compared with only 5.16% of background sequences, representing a 5.07-fold enrichment (*p* < 1 × 10^−55^). Together, these findings identify ATF3 as a functionally distinct and highly enriched transcription factor within UVB-remodeled chromatin regions, positioning it as a key epigenetic regulator opposing AP-1-driven oncogenic inflammation and malignant transformation.

### 3.3. The Transcriptional Shift in Keratinocytes Mediated by UVB-Irradiation

**Figure 3:**
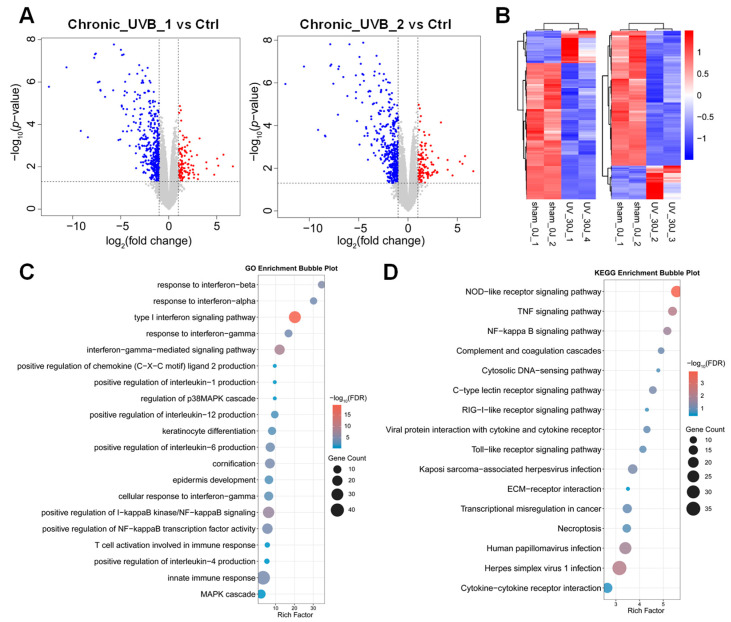
RNA-seq reveals transcriptomic and inflammatory changes in UVB-irradiated HaCaT cells. (**A**) Volcano plots of DEGs in two chronic UVB irradiation groups versus control. The vertical dashed lines represent log_2_(fold change) = ±1, and the horizontal dashed line corresponds to −log_10_(*p*-value) = 1, serving as cut-offs for screening differentially expressed genes. (**B**) Hierarchical clustering heatmap of DEGs, showing clear separation between control and UVB-irradiated groups. (**C**) GO enrichment bubble plot of DEGs, highlighting interferon and inflammatory pathways. (**D**) KEGG enrichment bubble plot of DEGs, emphasizing core inflammatory and oncogenic signaling pathways.

To compare gene expression profiles between control and chronic UVB-irradiated cells, we performed RNA-seq on the same samples as those used for ATAC-seq. The sequencing coverage and quality statistics of RNA-seq data are summarized in Supplementary File S2. A total of 291,720,286 raw reads were generated from six samples, yielding 266,697,676 clean reads after filtering, with over 96% of bases reaching Q30 (Table S2). We identified 32,771 genes, mostly mapped to coding sequence (CDS) regions (Figure S2A). Gene expression was normalized by RPKM, and biological replicates showed high intra-group correlation (Figure S2B,C). With thresholds of |log2 Fold change| ≥ 1 and q < 0.05, we identified 1136 DEGs, including 264 up and 872 downregulated genes (Figure 3A and Figure S2D). Hierarchical clustering heat map of DEGs confirmed consistent expression patterns within each group (Figure 3B).

To decipher the transcriptomic regulatory landscapes underlying chronic UVB-induced skin carcinogenesis, we performed Gene Ontology (GO) and Kyoto Encyclopedia of Genes and Genomes (KEGG) pathway enrichment analyses on 1136 DEGs between unirradiated and chronic UVB-irradiated groups. GO bubble plots revealed that the most significantly enriched pathways were predominantly associated with type I interferon (IFN) signaling, antiviral responses, and pro-inflammatory cascades, including type I interferon signaling pathway, response to interferon-α/β/γ, positive regulation of NF-κB signaling, and positive regulation of interleukin-6 production (Figure 3C). Type I IFN signaling, the top-enriched pathway, is critical for DNA damage repair and immune surveillance in keratinocytes, its dysregulation during chronic UVB exposure impairs tumor suppression and promotes immune escape in keratinocyte carcinoma [24]. These innate immune pathways bridge UVB-induced DNA damage to chronic inflammation, creating a feedforward loop that drives tumor initiation and progression [25]. We also observed robust enrichment of keratinocyte differentiation and epidermal development-related signaling cascades. Specifically, all oncogenic perturbations converge to dismantle the tightly regulated proliferation–differentiation hierarchy that underpins normal epidermal stratification, trapping epidermal progenitor cells in an immature, hyperproliferative state with unlimited replicative potential [26]. KEGG enrichment further confirmed the activation of core inflammatory and oncogenic pathways, with top-ranked terms including NOD-like receptor signaling pathway, TNF signaling pathway, NF-κB signaling pathway, and Toll-like receptor signaling pathway (Figure 3D). Notably, NF-κB signaling was simultaneously enriched in both GO and KEGG analyses of RNA-seq data, consistent with the elevated expression of p-p65 and p-IκBα within the NF-κB cascade (Figure 1F), which further validates the reliability of chronic UVB-induced malignant transformation model in HaCaT cells. In addition, the NF-κB and MAPK cascades, central to UVB-induced inflammatory responses, drive the production of pro-tumorigenic cytokines and sustain a pro-proliferative, anti-apoptotic microenvironment, accelerating malignant transformation [27,28]. Collectively, these results demonstrate that chronic UVB exposure reprograms the transcriptome of HaCaT cells toward a pro-inflammatory, pro-tumorigenic state, which are responsible for malignant transformation.

### 3.4. Integrated ATAC-Seq and RNA-Seq Analysis Identifies ATF3 as a Key Transcription Factor of Chronic UVB-Induced Keratinocyte Malignant Transformation

**Figure 4:**
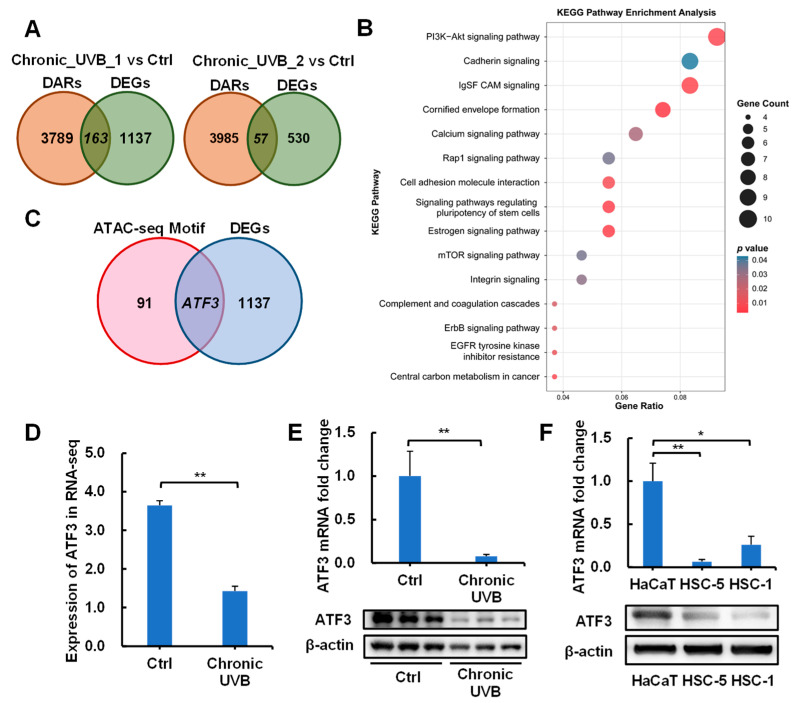
Integrated omics analysis identifies ATF3 as a key transcription factor in UVB-induced malignant transformation. (**A**) Venn diagrams of DARs and DEGs in two chronic UVB groups. (**B**) KEGG enrichment of overlapping DAR-DEGs. (**C**) Venn diagram illustrating the intersection of ATAC-seq-derived transcription factor motifs and DEGs, identifying ATF3 as a core candidate regulator. (**D**) ATF3 expression levels from RNA-seq data, demonstrating significantly reduced ATF3 expression in the chronic UVB group compared with the control group. (**E**) qPCR and Western blot validation of ATF3 downregulation in chronic UVB-irradiated HaCaT cells. (**F**) qPCR and Western blot analysis of ATF3 expression in normal HaCaT cells and cSCC cell lines. * *p* < 0.05, ** *p* < 0.01.

Independent analyses of ATAC-seq and RNA-seq datasets separately uncovered UVB-triggered chromatin remodeling and transcriptional perturbations in HaCaT cells, which underscores the necessity of integrated multi-omics profiling. Therefore, integrated analysis of ATAC-seq and RNA-seq data was performed to identify core genes coordinately regulated at the epigenetic and transcriptional levels during chronic UVB-induced malignant transformation of HaCaT cells. Integration of DARs and DEGs from the two groups yielded 163 and 57 overlapping genes, respectively (Figure 4A). The intersection genes from both groups were subjected to KEGG pathway enrichment analysis. The most significantly enriched pathways included PI3K-Akt signaling, cadherin signaling, IgSF CAM signaling, cornified envelope formation, and calcium signaling, alongside key oncogenic pathways such as central carbon metabolism in cancer and EGFR inhibitor resistance (Figure 4B). To further identify key transcription factors implicated in chronic UVB-induced keratinocyte tumorigenesis, overlapping analysis was performed between transcription factor genes predicted via ATAC-seq motif scanning and RNA-seq-derived DEGs. Multiple candidate transcription factors were obtained from the intersection set, among which ATF3 was prioritized as the core regulatory candidate for subsequent mechanistic investigation (Figure 4C). Distinct from other enriched transcription factors of the same protein family including c-FOS, BATF3, Fra1 and JunB, ATF3 was the sole candidate that simultaneously showed significant differential chromatin enrichment in ATAC-seq motif analysis and altered gene expression in RNA-seq profiling, while the expression levels of the remaining family members remained unaltered under chronic UVB treatment. Additionally, the mTOR inhibitor sirolimus can reverse ATF3 overexpression in healthy keratinocytes yet fails to suppress ATF3 in established cSCC cell lines, confirmed the oncogenic role of ATF3 in cutaneous malignant progression [29], providing solid rationale for focusing on ATF3 as the primary research target. Consistent with the transcriptomic profiling data, qPCR and Western blot detection confirmed markedly reduced ATF3 mRNA and protein abundance in chronic UVB-irradiated HaCaT cells relative to unirradited HaCaT cells (Figure 4D,E). Likewise, endogenous ATF3 expression was significantly suppressed in two human cutaneous squamous cell carcinoma cell lines HSC-1 and HSC-5, when compared with normal HaCaT keratinocytes (Figure 4F). Collectively, these results suggest that loss of ATF3 may contribute to UVB-induced tumorigenesis.

### 3.5. ATF3 Suppressed Chronic UVB-Induced Malignant Transformation of Keratinocytes

**Figure 5:**
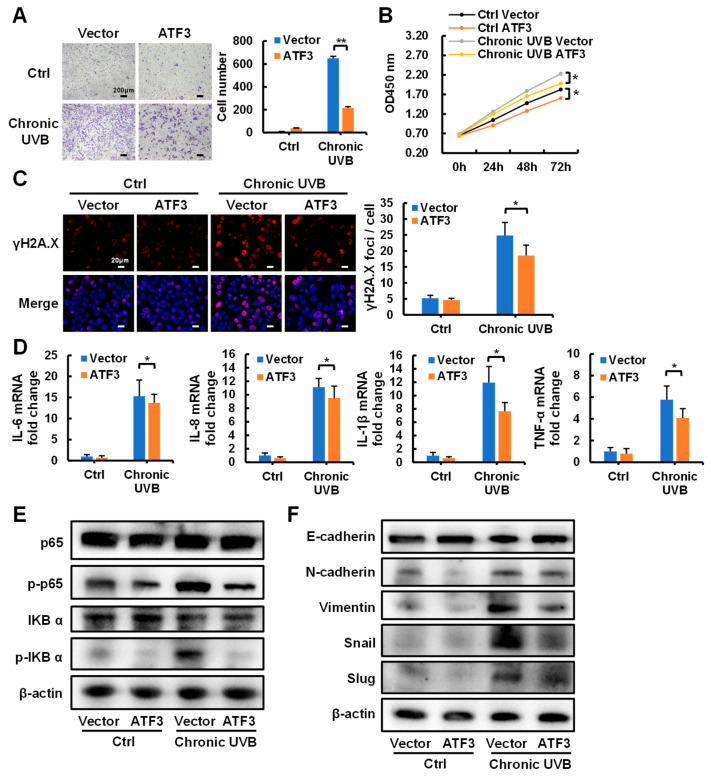
ATF3 acts as a tumor suppressor in chronic UVB-irradiated HaCaT cells. (**A**) Representative bright-field images and quantitative statistics of Transwell migration assays show ATF3 inhibits migration of chronic UVB-irradiated HaCaT cells, migrated cell counts were quantified and plotted in the right panel. (**B**) CCK-8 proliferation assay shows that ATF3 overexpression significantly enhances proliferation of chronic UVB-irradiated HaCaT cells. (**C**) Representative immunofluorescence images and quantitative quantification of γH2A.X nuclear foci in vector and ATF3-overexpressing HaCaT cells under control or chronic UVB conditions. Nuclei were counterstained with DAPI (blue). (**D**) Transcript levels in vector and ATF3-overexpressing HaCaT cells under basal or chronic UVB irradiation. (**E**) Western blot analysis of total p65, p-p65, total IκBα and p-IκBα to evaluate NF-κB pathway activation; β-actin served as the loading control. (**F**) Immunoblot detection of EMT biomarkers including epithelial marker E-cadherin, mesenchymal markers N-cadherin, Vimentin, Snail and Slug. β-actin was used for equal protein loading normalization. All data are presented as mean ± SD from three independent biological replicates. * *p* < 0.05, ** *p* < 0.01.

Prior multi-omics screening identified ATF3 as a key transcription factor regulating UVB-induced malignant transformation, highlighting the critical value of subsequent functional validation. To elucidate the role of ATF3 in chronic UVB-induced malignant transformation of keratinocytes, a series of cellular functional validation assays were carried out. Transfection of three independent ATF3-targeting siRNAs into control or chronic UVB-irradiated HaCaT cells significantly reduced ATF3 mRNA and protein levels, and the transfection with ATF3-overexpressing plasmids markedly increased its expression, which validated the efficiency of transfection for modulating ATF3 expression (Figure S3A,B). Transwell migration assay demonstrated that ATF3 knockdown significantly promoted cell migration in both groups, particularly in UVB-irradiated cells, whereas ATF3 overexpression inhibited migration, with UVB-irradiated cells showing reduced migratory capacity compared to unirradiated cells (Figure 5A and Figure S4A). Cell proliferation dynamics were quantified via CCK-8 assays to characterize ATF3-dependent growth regulation in keratinocytes. ATF3 knockdown significantly enhanced cell proliferation in both unirradiated and UVB-irradiated HaCaT cells, with a more pronounced effect in UVB-irradiated cells. Conversely, ATF3 overexpression significantly suppressed cell proliferation, with UVB-irradiated cells exhibiting weaker proliferative activity than unirradiated cells (Figure 5B and Figure S4B).

Genomic DNA damage represents a core initiating event driving UVB-mediated keratinocyte malignant transformation; thus, immunofluorescence staining targeting γH2A.X, a canonical marker of DNA double-strand breaks, was used to quantify irradiation-elicited genomic damage. Chronic UVB irradiation triggered robust formation of nuclear γH2A.X foci. ATF3 overexpression markedly reduced the number of γH2A.X-positive foci in UVB-irradiated HaCaT cells (Figure 5C). In contrast, ATF3 knockdown drastically elevated γH2A.X foci abundance specifically under chronic UVB stress, with no evident changes detected in unirradiated cells following ATF3 depletion (Figure S4C,D). Persistent inflammatory signaling facilitates the progression of UVB-induced precancerous lesions; therefore, qPCR was conducted to clarify whether ATF3 governs the transcription of pro-inflammatory cytokines upon chronic UVB irradiation. qPCR quantification revealed robust transcriptional upregulation of IL-6, IL-8, IL-1β and TNF-α transcripts upon chronic UVB irradiation. ATF3 overexpression significantly repressed the transcriptional induction of all four inflammatory cytokines in UVB-irradiated HaCaT cells (Figure 5D). Reciprocally, ATF3 deficiency further amplified UVB-triggered upregulation of these pro-inflammatory transcripts, while ATF3 knockdown exerted negligible effects on basal cytokine expression without UVB irradiation (Figure 5E).

To further delineate the pivotal function of ATF3 during chronic UVB-triggered keratinocyte malignant transformation, we interrogated NF-κB signaling and epithelial–mesenchymal transition (EMT), two canonical pathways tightly implicated in skin tumorigenesis and malignant progression. Immunoblot assay revealed that ATF3 overexpression significantly increased the protein abundance of phosphorylated p65 (p-p65) and phosphorylated IκBα (p-IκBα) under chronic UVB exposure, whereas ATF3 knockdown markedly attenuated the levels of these phosphorylated NF-κB signaling mediators (Figure 5E and Figure S4F). For EMT phenotypes, ATF3 overexpression restored the epithelial marker E-cadherin and simultaneously suppressed mesenchymal markers N-cadherin, Vimentin, Slug and Snail in UVB-treated keratinocytes (Figure 5F). Conversely, knockdown ATF3 further diminished E-cadherin expression while robustly upregulating all detected mesenchymal EMT transcription factors and structural proteins (Figure S4G). Collectively, these findings confirm that ATF3 governs the activation of NF-κB signaling and shapes the expression profile of core EMT markers in the context of chronic UVB-induced keratinocyte malignant transformation.

Taken together, these functional phenotypic assays demonstrate that ATF3 antagonizes multiple malignant features induced by chronic UVB irradiation in keratinocytes, including aberrant proliferation, enhanced migration, accumulated genomic DNA damage, hyperactivated pro-inflammatory signaling cascades, dysregulated NF-κB activation, and altered expression of core EMT biomarkers, thereby suppressing UVB-induced malignant transformation.

### 3.6. Screening and Identification of ATF3 Target Genes in Chronic UVB-Induced Keratinocyte Malignant Transformation

**Figure 6:**
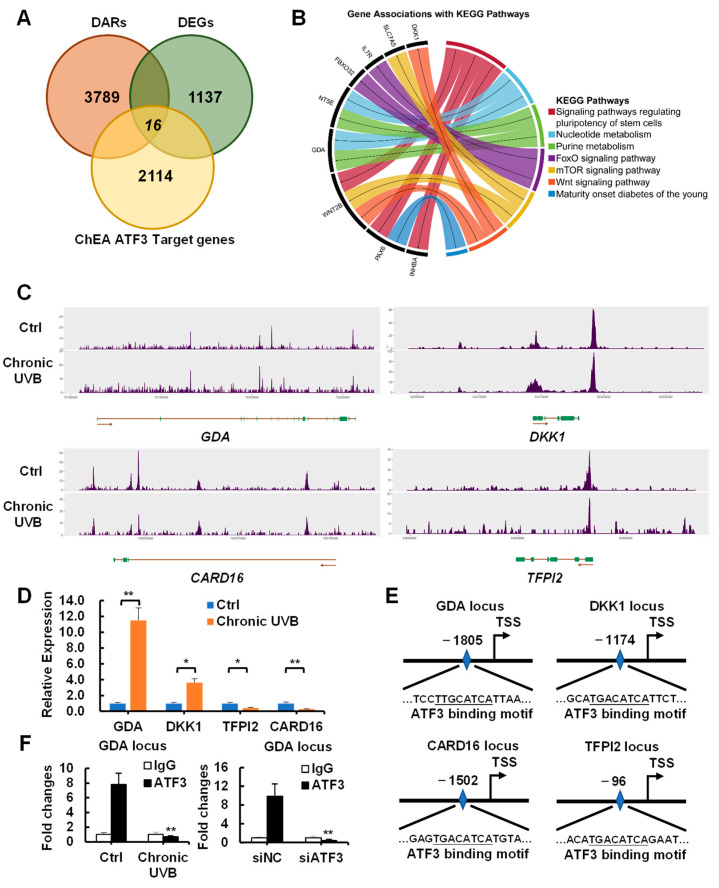
Identification of ATF3-regulated downstream target genes in chronic UVB-induced keratinocyte malignant transformation. (**A**) Venn diagram of ChEA ATF3 Targets, DARs and DEGs, yielding 16 overlapping candidate genes. (**B**) Circos diagram displaying KEGG pathway enrichment results of the 16 intersecting genes, with colored arcs representing distinct enriched oncogenic signaling pathways. (**C**) Genome browser tracks illustrating chromatin accessibility signals at the *GDA*, *DKK1*, *CARD16* and *TFPI2* gene loci in control and chronic UVB-irradiated HaCaT cells. Elevated signal intensity indicates increased chromatin openness. The arrow below marks the transcription direction of the indicated genes. (**D**) qPCR quantification of relative mRNA expression levels of GDA, DKK1, TFPI2 and CARD16 in control and chronic UVB-treated HaCaT cells. Data are presented as mean ± SD; * *p* < 0.05, ** *p* < 0.01 versus the control group. (**E**) Schematic annotation of ATF3 canonical binding motifs within the promoter regions of GDA, DKK1, CARD16 and TFPI2, marking the exact base-pair distance of each motif relative to the TSS, together with corresponding underlying motif DNA sequences. (**F**) ChIP-qPCR analysis measuring relative ATF3 binding fold enrichment at the GDA promoter motif locus. Left: comparison between control and chronic UVB HaCaT cells; right: comparison between siNC negative control and ATF3 siRNA-transfected HaCaT cells. Normal rabbit IgG served as background control. Data represent mean ± SD; * *p* < 0.05, ** *p* < 0.01.

Given ATF3 functions as a master transcription factor that exerts tumor-suppressive activity via binding to downstream target genes, we sought to identify ATF3 downstream targets in UVB-induced HaCaT malignant transformation, we intersected ChIP-X Enrichment Analysis (ChEA) database predictions with DARs and DEGs, yielding 16 overlapping genes (Figure 6A). KEGG pathway enrichment analysis of these 16 overlapping genes revealed significant enrichment in critical oncogenic pathways, including signaling pathways regulating pluripotency of stem cells, Wnt signaling pathway, mTOR signaling pathway, FoxO signaling pathway, and purine metabolism (Figure 6B), which collectively drive cell proliferation, stemness maintenance, metabolic reprogramming, and survival during UVB-induced skin carcinogenesis. Among the 16 overlapping genes, we prioritized four tumorigenesis-relevant candidates, including *GDA*, *DKK1*, *CARD16* and *TFPI2*. This screening criterion was based on motif alignment analysis of promoter sequences covering the region 2000 bp upstream of each gene’s transcription start site (TSS). Unique canonical ATF3 binding DNA motifs were exclusively detected within the promoter regions of these four genes [15]. The ATF3 consensus motif sequence TTGCATCA was identified at the *GDA* promoter locus, while the conserved binding motif TGACATCA was detected in the promoter sequences of *DKK1*, *CARD16* and *TFPI2*; however, no canonical ATF3 binding motifs were identified in the remaining 12 intersecting genes.

IGV (Integrative Genomics Viewer) genome browser tracks further revealed divergent changes in chromatin accessibility at the four gene loci following chronic UVB exposure (Figure 6C). Significant increases in chromatin accessibility are observed at the *GDA* and *DKK1* promoter regions in response to sustained UVB irradiation, whereas the chromatin openness of the *CARD16* and *TFPI2* promoters is substantially reduced. This divergent chromatin remodeling landscape stems from the dual regulatory capacity of ATF3, which exerts context-dependent transcriptional repression or activation on its downstream targets. Disassociation of ATF3 alleviates basal transcriptional repression and promotes local chromatin decompaction, which consequently elevates the transcriptional activity of GDA and DKK1. Conversely, reduced ATF3 occupancy at their promoters abrogates ATF3-mediated transcriptional activation under chronic UVB treatment, inducing chromatin condensation and eventually suppresses the transcription of CARD16 and TFPI2. Consistent with RNA-seq profiling results, the mRNA expression levels of GDA and DKK1 were significantly upregulated in chronic UVB-treated HaCaT cells relative to unirradited HaCaT cells, while CARD16 and TFPI2 transcripts were markedly downregulated after long-term UVB irradiation (Figure 6D).

To verify the physical interaction between ATF3 protein and the predicted ATF3-binding promoter motifs of *GDA*, *DKK1*, *CARD16* and *TFPI2*, promoter motif scanning was performed using the UCSC Genome Browser (http://genome.ucsc.edu/, accessed on 28 May 2026) and rVista (https://rvista.dcode.org/, accessed on 28 May 2026) (Figure 6E). Subsequent ChIP-qPCR assays confirmed that ATF3 enrichment at the GDA promoter binding site was significantly reduced in chronic UVB-exposed HaCaT cells compared with the control group. Consistently, siRNA-mediated ATF3 knockdown in unirradited HaCaT cells also triggered a pronounced decline in ATF3 occupancy at the GDA promoter locus (Figure 6F). In contrast, no statistically significant alterations in ATF3 binding enrichment were detected at the DKK1, CARD16 and TFPI2 promoter motifs, either following chronic UVB irradiation or ATF3 silencing (Figure S5).

Collectively, multi-omics intersection screening, chromatin accessibility profiling, transcriptional quantification and ChIP-qPCR binding validation demonstrate that ATF3 directly binds the canonical motif within the *GDA* promoter to transcriptionally repress GDA expression under physiological conditions. Chronic UVB exposure diminishes ATF3 recruitment to the *GDA* promoter, relieving transcriptional repression and increasing chromatin accessibility to drive GDA overexpression, which contributes to UVB-induced keratinocyte malignant transformation.

## 4. Discussion

The malignant transformation of normal keratinocytes into invasive KC is a multistep process characterized by dynamic epigenetic and transcriptional alterations, as well as dysregulated signaling pathways [30]. Elucidating the molecular mechanisms governing this transformative process is critical for developing effective preventive and therapeutic strategies, as advanced KC remains associated with significant morbidity and limited treatment options [31]. Despite extensive research on UVB-induced skin carcinogenesis, the epigenetic regulatory networks underlying keratinocyte malignant transformation remain incompletely understood, highlighting the need for integrated multi-omics approaches to dissect these complex processes.

In this study, we employed an integrative approach combining ATAC-seq and RNA-seq to systematically profile chromatin accessibility, transcriptomic changes, and signaling pathway alterations during chronic UVB-induced malignant transformation of normal HaCaT keratinocytes. Chromatin accessibility is a fundamental epigenetic feature that directly modulates gene expression by governing transcription factor binding to regulatory regions, aberrant chromatin dynamics are increasingly recognized as key drivers of tumorigenesis [32]. ATAC-seq has emerged as a powerful tool for mapping genome-wide open chromatin landscapes, while RNA-seq quantifies corresponding gene expression changes [33]. The integration of these two modalities overcomes the limitations of single-omics analyses, enabling the identification of functionally relevant regulatory elements, their associated TFs, and target genes [34]. Our integrated analysis successfully captured dynamic changes in chromatin accessibility, gene expression, and signaling pathways during the transition from normal keratinocytes to malignant cells, leading to the identification of ATF3 as a critical regulator of this process. Functional experiments further demonstrated that ATF3 deletion promotes tumorigenesis, and targeted regulation of ATF3 may delay or inhibit UVB-induced KC initiation.

Motif enrichment analysis of DARs from ATAC-seq revealed predominant enrichment of AP-1 family TFs, including c-Fos, Fra1, BATF3, and JunB, which is consistent with previous reports implicating AP-1 family members in UVB-induced skin carcinogenesis [35]. As key stress-responsive transcription factors, AP-1 complexes are rapidly activated following UVB exposure and orchestrate a cascade of pro-tumorigenic transcriptional programs. c-Fos and JunB heterodimers drive cell cycle progression, and extracellular matrix remodeling, thereby facilitating keratinocyte hyperproliferation and invasive behavior [19]. Fra1 overexpression stabilizes AP-1-driven gene expression programs linked to MMP production and metastatic potential [20]. BATF modulates inflammatory cytokine secretion and reshapes the tumor immune microenvironment by regulating IL-6 and IL-8 expression, further promoting a pro-tumorigenic milieu [21]. Furthermore, AP-1 crosstalk with NF-κB amplifies oncogenic signaling, establishing it as a critical target for chemopreventive strategies against non-melanoma skin cancer [35]. Strikingly, among these AP-1 family members, only ATF3 showed significant overlap with DEGs from RNA-seq, indicating its unique functional role in chronic UVB-induced keratinocyte malignant transformation. This observation is particularly notable given that previous studies have demonstrated upregulation of ATF3 following acute, single-dose UV exposure, where it mediates protective cellular responses such as DNA repair and apoptosis [36]. In contrast, our transcriptomic data uncovered marked suppression of ATF3 in keratinocytes under chronic UVB irradiation, representing a novel observation that highlights context-dependent regulation of ATF3 in acute stress versus chronic carcinogenic exposure.

Enrichment analyses of DEGs revealed robust activation of inflammatory pathways following chronic UVB irradiation. Gene Ontology terms included type I interferon signaling, responses to interferons α/β/γ, positive regulation of NF-κB activity, and interleukin-6 production. Corresponding KEGG pathways comprised viral protein-cytokine interactions, Toll-like receptor signaling, TNF signaling, NOD-like receptor signaling, and NF-κB signaling. These findings align with previous studies emphasizing chronic inflammation as a key driver of UVB-induced skin carcinogenesis [37,38]. Functional experiments confirmed that ATF3 acts as a tumor suppressor in UVB-induced KC: ATF3 overexpression markedly restrained keratinocyte proliferation, weakened migratory potential, alleviated UVB-evoked DNA damage, reduced pro-inflammatory cytokine secretion, suppressed NF-κB activation and reversed abnormal EMT biomarker expression, whereas knockdown of ATF3 exacerbated all these oncogenic phenotypes. This is consistent with emerging evidence supporting a tumor-suppressive role of ATF3 in various cancers, including skin tumors [15,16]. ATF3 has been shown to be downregulated in stromal cells of photo-damaged premalignant lesions [39], and ATF3 knockout mice develop more aggressive chemically induced skin tumors [40], further supporting its tumor-suppressive function. Our findings extend these observations by demonstrating that ATF3 downregulation during chronic UVB irradiation promotes keratinocyte malignant transformation, highlighting its critical role in this specific oncogenic context.

To dissect how ATF3 mediates tumor suppression, we intersected ATF3 target predictions from ChEA with DARs and DEGs, identifying 16 candidate downstream target genes. Promoter motif analysis (−2000 bp relative to the TSS) further screened out four genes possessing canonical ATF3-binding sequences, including *GDA*, *DKK1*, *CARD16*, and *TFPI2*, while no valid ATF3 motifs were detected in the remaining candidates. Specifically, GDA and DKK1 exhibited increased promoter chromatin accessibility and upregulated mRNA expression, whereas CARD16 and TFPI2 displayed reduced chromatin openness and transcriptional downregulation, which was consistent with transcriptomic data. Notably, upregulated in UVB-irradiated HaCaT cells, GDA mediates keratinocyte senescence via uric acid metabolism and promotes melanogenesis through the ABCG2-PDZK1-p38 MAPK axis, thereby contributing to skin tumorigenesis [41]. High DKK1 expression in head and neck squamous cell carcinoma (HNSCC) drives radio- and immunotherapy resistance via PI3K-Akt pathways and immunosuppressive microenvironment modulation, making it a promising target for combined radioimmunotherapy [42]. Additionally, CARD16 overexpression drives tumorigenesis and invasion by downregulating P21 to activate CDK2, which promotes FOXO1 phosphorylation and degradation [43]. TFPI2 knockdown markedly boosts melanoma cell proliferation and invasive capacity to drive aggressive tumor progression [44]. Subsequent ChIP-qPCR validation confirmed that chronic UVB irradiation significantly reduced ATF3 binding enrichment at the GDA promoter. Consistently, siRNA-mediated ATF3 knockdown also diminished ATF3 occupancy at the GDA locus. However, ATF3 binding to DKK1, CARD16 and TFPI2 showed no significant alterations upon either UVB irradiation or ATF3 depletion, indicating that GDA acts as the direct transcriptional target of ATF3. As a transcription factor, ATF3 relies on chromatin accessibility to mediate the transcription of downstream target genes, thereby modulating the initiation and progression of cutaneous tumors [45]. Combined with ChIP-qPCR evidence verifying *GDA* as a direct downstream target of ATF3 throughout chronic UVB-induced malignant transformation of HaCaT cells, we hypothesize that ATF3 transcriptionally regulates GDA expression by remodeling chromatin accessibility at the GDA locus. Collectively, chronic UVB irradiation induces the downregulation of endogenous ATF3, thereby increasing chromatin accessibility and elevating GDA expression to facilitate keratinocyte malignant transformation.

Nevertheless, several limitations of the present study should be acknowledged. All phenotypic and molecular validations were performed using the HaCaT cell line, an immortalized keratinocyte cell line rather than primary human epidermal keratinocytes. The transformed characteristics and epigenetic responses of immortalized cells may not fully recapitulate the biological behaviors of primary keratinocytes within the native human skin microenvironment. However, the long-term chronic UVB exposure model spanning 16 weeks requires sustained serial passaging of keratinocytes throughout the experimental period, primary epidermal keratinocytes inevitably undergo severe senescence or cell death under prolonged UVB irradiation and cannot sustain such long-term culture cycles. Under this experimental constraint, the immortalized HaCaT cell line is an indispensable tool to guarantee stable cell viability over the 16-week continuous transformation induction process. In addition, all conclusions in this study are derived from in vitro cell models. Further in vivo skin tumorigenesis animal models are required to validate the regulatory role of ATF3 in chronic UVB-induced skin malignant transformation and verify the translational potential of ATF3-targeted intervention.

## 5. Conclusions

In summary, our study demonstrates that chronic UVB irradiation downregulates ATF3, leading to chromatin remodeling, activation of inflammatory signaling pathways, and overexpression of oncogenic target genes, thereby promoting keratinocyte malignant transformation. Notably, through multi-omics screening and ChIP-qPCR validation, we further identified and confirmed *GDA* as the direct downstream target gene of ATF3 in this UVB-induced tumorigenic process. A key strength of our study is the use of an integrated ATAC-seq and RNA-seq strategy, which efficiently pinpointed ATF3 as a pivotal tumor suppressor and clarified its regulatory axis targeting *GDA*, thereby providing novel epigenetic and transcriptional insights into the pathogenesis of UVB-induced keratinocyte carcinoma. This multi-omics combinatorial strategy provides a reliable reference for future mechanistic studies on skin cancer initiation and progression. Limitations include the use of an in vitro HaCaT cell model, and future in vivo animal experiments are required to further validate the ATF3-GDA regulatory network. Overall, our findings highlight ATF3 and its direct target *GDA* as promising preventive and therapeutic biomarkers for keratinocyte carcinoma, offering potential strategies to reduce the occurrence and progression of UVB-induced keratinocyte carcinoma.

## Figures and Tables

**Table 1:** Primer sequences used for qPCR.

Genes	Forward Primer (5′ → 3′)	Reverse Primer (5′ → 3′)
*ATF3*	CGCTGGAATCAGTCACTGTCAG	CTTGTTTCGGCACTTTGCAGCTG
*IL-6*	AGACAGCCACTCACCTCTTCAG	TTCTGCCAGTGCCTCTTTGCTG
*IL-8*	GAGAGTGATTGAGAGTGGACCAC	CACAACCCTCTGCACCCAGTTT
*IL-1β*	CCACAGACCTTCCAGGAGAATG	GTGCAGTTCAGTGATCGTACAGG
*TNF-α*	CTCTTCTGCCTGCTGCACTTTG	ATGGGCTACAGGCTTGTCACTC
*GDA*	CACACTGACTCATCTCTGCTCC	CGATTCCTCAGTGGTCTCCTTG
*DKK1*	GGTATTCCAGAAGAACCACCTTG	CTTGGACCAGAAGTGTCTAGCAC
*CARD16*	ATTCCGAAAGGGGCACAGGCAT	TCTGCCTTCTGGGCTTGAGCAT
*TFPI2*	TACTGGCTGTGGAGGGAATGAC	CGGATTCTACTGGCAAAGCGAAG
*GAPDH*	GTCTCCTCTGACTTCAACAGCG	ACCACCCTGTTGCTGTAGCCAA

**Table 2:** Primer sequences used for ChIP-qPCR.

Genes	Forward Primer (5′ → 3′)	Reverse Primer (5′ → 3′)
*GDA-ATF3*	TGGGCCAGCTCATTCCTTCTTT	TGCCTCCTTGATGTCTCTGTGT
*DKK1-ATF3*	CCCACCACCCATCTCACCAAA	GGGACCACGCAATACCCTTTCT
*CARD16-ATF3*	CATGCCACTGCACTCCAACCT	TGAACCACTGTTCCCTTGACCC
*TFPI2-ATF3*	TGACAGCATTTCCAGATCAGGC	ACACTTGCTCAAAGTGGGTT

## Data Availability

The ATAC-seq and RNA-seq data generated in this study are publicly available in the Sequence Read Archive (SRA) under the accession number [PRJNA1453714] (https://www.ncbi.nlm.nih.gov/sra/PRJNA1453714, accessed on 14 April 2026). All other data supporting the findings of this study are available within the article and its Supplementary Materials. Any additional raw or processed data are available from the corresponding author upon reasonable request.

## Supplementary Materials

The following supporting information can be downloaded at https://zenodo.org/records/21253988 (accessed on 8 July 2026), File S1: Uncropped images of Western blotting; File S2: Sequencing coverage and quality statistics; Figure S1: Sample correlation and global chromatin accessibility analysis of chronic UVB-irradiated HaCaT cells; Figure S2: RNA-seq quality control and DEG analysis of chronic UVB-irradiated HaCaT cells; Figure S3: Efficiency of ATF3 knockdown and overexpression in HaCaT cells; Figure S4: ATF3 knockdown aggravates UVB-induced malignant phenotypes of HaCaT cells; Figure S5: ChIP-qPCR analysis of ATF3 binding to the promoter regions of DKK1, CARD16 and TFPI2; Table S1: ATAC-seq data quality control; Table S2: mRNA-seq data quality control.

## Abbreviations

The following abbreviations are used in this manuscript:

ATAC-seq	Assay for Transposase-Accessible Chromatin-sequencing
ATF3	Activating Transcription Factor 3
CAFs	Cancer-associated fibroblasts
CDS	Coding sequence
ChEA	ChIP-X enrichment analysis
cSCC	Cutaneous Squamous Cell Carcinoma
DARs	Differentially Accessible Regions
DEGs	Differentially Expressed Genes
EMT	Epithelial–Mesenchymal Transition
GDA	Guanine deaminase
GO	Gene Ontology
HNSCC	Head and Neck Squamous Cell Carcinoma
KC	Keratinocyte Carcinoma
KEGG	Kyoto Encyclopedia of Genes and Genomes
MMPs	Matrix Metalloproteinases
NMSCs	Non-melanoma skin cancers
ROS	Reactive Oxygen Species
UVB	Ultraviolet B
